# A systematic review and meta-analysis of trypanosome prevalence in tsetse flies

**DOI:** 10.1186/s12917-017-1012-9

**Published:** 2017-04-13

**Authors:** Reta D. Abdi, Getahun E. Agga, Weldegebrial G. Aregawi, Merga Bekana, Thomas Van Leeuwen, Vincent Delespaux, Luc Duchateau

**Affiliations:** 1 Department of Clinical studies, College of Veterinary Medicine and Agriculture, Addis Ababa University, Bishoftu, Oromia Ethiopia; 2 Department of Animal Science, Institute of Agriculture, University of Tennessee, 2506 River Drive, Knoxville, USA; 3 U.S. Department of Agriculture, Agricultural Research Service, Food Animal Environmental Systems Research Unit, Bowling Green, Kentucky, USA; 4 Werer Agricultural Research Center, Ethiopian Institute of Agricultural Research, Afar, Ethiopia; 5 Department of Crop Protection, Faculty of Bioscience Engineering, Gent University, Ghent, Belgium; 6 Faculty of Sciences and Bioengineering Sciences, Vrije Universiteit Brussel, Brussels, Belgium; 7 Department of Comparative Physiology and Biometrics, Faculty of Veterinary Sciences, Gent University, Ghent, Belgium

**Keywords:** Meta-regression, Systematic review, Glossina, Trypanosome infection prevalence, Diagnostic methods

## Abstract

**Background:**

The optimisation of trypanosomosis control programs warrants a good knowledge of the main vector of animal and human trypanosomes in sub-Saharan Africa, the tsetse fly. An important aspect of the tsetse fly population is its trypanosome infection prevalence, as it determines the intensity of the transmission of the parasite by the vector. We therefore conducted a systematic review of published studies documenting trypanosome infection prevalence from field surveys or from laboratory experiments under controlled conditions. Publications were screened in the Web of Science, PubMed and Google Scholar databases. Using the four-stage (identification, screening, eligibility and inclusion) process in the PRISMA statement the initial screened total of 605 studies were reduced to 72 studies. The microscopic examination of dissected flies (dissection method) remains the most used method to detect trypanosomes and thus constituted the main focus of this analysis. Meta-regression was performed to identify factors responsible for high trypanosome prevalence in the vectors and a random effects meta-analysis was used to report the sensitivity of molecular and serological tests using the dissection method as gold standard.

**Results:**

The overall pooled prevalence was 10.3% (95% confidence interval [CI] = 8.1%, 12.4%) and 31.0% (95% CI = 20.0%, 42.0%) for the field survey and laboratory experiment data respectively. The country and the year of publication were found to be significantly factors associated with the prevalence of trypanosome infection in tsetse flies. The alternative diagnostic tools applied to dissection positive samples were characterised by low sensitivity, and no information on the specificity was available at all.

**Conclusion:**

Both temporal and spatial variation in trypanosome infection prevalence of field collected tsetse flies exists, but further investigation on real risk factors is needed how this variation can be explained. Improving the sensitivity and determining the specificity of these alternative diagnostic tools should be a priority and will allow to estimate the prevalence of trypanosome infection in tsetse flies in high-throughput.

**Electronic supplementary material:**

The online version of this article (doi:10.1186/s12917-017-1012-9) contains supplementary material, which is available to authorized users.

## Background

*Glossina* species (commonly known as tsetse flies) are the major vectors of several *Trypanosoma* species, the causative agents of animal and human African trypanosomosis, also called Nagana and sleeping sickness, respectively [1–3]. Once established in the tsetse fly, trypanosomes undergo a developmental cycle within the tsetse fly with varying complexity depending on the species [4]. The infected tsetse fly then transmits the parasite to diverse host species during its blood meal. Tsetse flies infest an area of about 10 million km^2^ comprising 38 sub-Saharan African countries [5]. The disease constitutes a major veterinary and medical burden affecting the life of millions of people. Within affected regions, the density of the vector and the prevalence of trypanosome infections in the host is attributed to complex interactions between and among humans, domestic livestock, wildlife, tsetse flies, trypanosomes and various economic and ecological factors [6, 7].

The prevalence of trypanosome infections in the tsetse flies is often a neglected parameter probably due to the intensive labour required for its evaluation. Integrating this parameter in a monitoring program allows however a more precise evaluation of the risk of being infected in a particular region.

Dissection of flies remains the most common technique for detecting the presence of trypanosomes. Although molecular and serological techniques are assumed to detect far higher levels of genetic diversity with a higher sensitivity [8], the performance of such tests has been reported to be unsatisfactory [9–13]. For instance, PCR failed to detect trypanosomes in dissection positive flies or vice versa, and tsetse fly samples negative by PCR were positive by fluorescent fragment length barcoding tests even allowing the discovery of new genotypes [14, 15].

The aim of this systematic review was to (i) synthesize the limited information on the trypanosome prevalence in tsetse flies, and (ii) assess the sensitivity of various diagnostic methods for the detection of trypanosomes in the tsetse flies using the dissection method as gold standard.

## Methods

### Search strategy and inclusion of studies

**Fig. 1:**
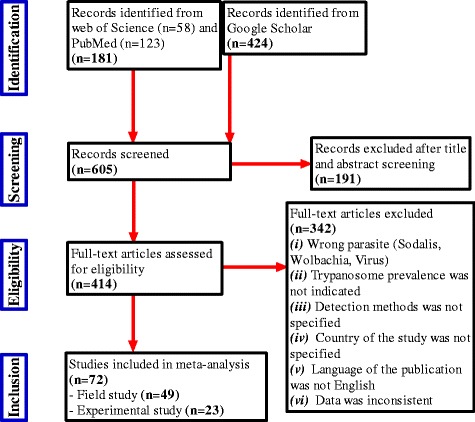
Flowchart detailing the number of studies excluded and included at each step for systematic review of the prevalence of trypanosome infection in tsetse flies

Publications were screened in the Web of Science, PubMed and Google Scholar databases. The last search was done on July the 20th 2015. The following Boolean parameter combinations and Medical Subject Headings terms were used: “Trypanosomes” and “infection rate” and “tsetse fly or *Glossina*”. The retrieved articles were then first screened by title and abstract by two independent readers. Any discrepancies were discussed until consensus. Selected articles were retained for further full text analysis. The inclusion criteria for further data extraction and meta-analysis were the presence of the following data: (i) tsetse species, (ii) study type (laboratory or field), (iii) location (country) of study, (iv) trypanosome detection method, (v) type of tsetse sample examined, (vi) number and type of fly samples, and (vii) number of samples positive for trypanosomes. A flow chart describing the number of articles retrieved, screened and included or rejected is presented in Fig. 1.

### Meta-analysis

All meta-analyses were performed with STATA Version 12 (StataCorp LP, College Station, Texas). The core analysis focused on the prevalence of trypanosome infections in the tsetse flies as assessed by the dissection technique. Analyses are done separately for data obtained from field surveys and laboratory experiments. In laboratory experiments, blood meals and external conditions are standardized and all feeding flies ingest parasites. In the field surveys, the prevalence of trypanosomes in the host and the parasitaemia will be the determining factors explaining the prevalence in flies. First, pooled estimates were calculated based on the random effects model [16, 17] with study as random effect. Results were presented by forest plots. Next, factors that could be associated with the prevalence of infection were considered using logistic meta-regression analysis [18, 19] with country, tsetse species and tsetse organ as categorical and year of publication as continuous fixed effects factors. Odds ratios with their 95% confidence interval (CI) were used as summary statistic and testing was done at the 5% significance level.

A second part of the meta-analysis assessed the sensitivity of the alternative diagnostic tests using the dissection method as a gold standard. The sensitivity was evaluated using the random effects model.

### Diagnostic tools to assess trypanosome infection in a tsetse fly

The most widely used method constitutes the dissection of tsetse flies and microscopic evaluation of the organs. It is cheap [20], but laborious, low in sensitivity and cannot differentiate mixed infections of trypanosome species and its different developmental stages in the fly [21]. The warm slide technique is occasionally used to assess the prevalence of trypanosomes in tsetse flies. Accordingly, tsetse flies are allowed to salivate (probe) on a warm slide, then, trypanosome examination is done by microscopy of the slide [22]. An alternative for parasite detection with higher sensitivity is the inoculation of dissolved organ contents of tsetse flies in rats or mice for xenodiagnosis. Its added merit is that field isolates from mammals or tsetse flies can be collected via rodent inoculation for further studies. Its disadvantage is that diagnosis is not immediate and that *T. vivax* and *T. simiae* do not infect rodents [23]. Tissue culture techniques (in vitro cultivation) can be another option using different culture media. Cultivation is widely done for species of the *Trypanosoma brucei* group. The culture method is vital as it can provide information on pathogen viability and susceptibility to drugs [23, 24]. Isoenzyme band pattern examination technique is also possible. In this technique, 10–20 enzymes extracts from the trypanosome cytoplasm common to nearly all trypanosome species are separated by native electrophoresis and visualized by native staining. It requires a minimum of 100 million trypanosomes to test positive [25, 26]. The dot-ELISA test is another option which is based on the preparation of suspensions of different organs of tsetse flies that are applied on nitrocellulose membranes. Trypanosome species-specific monoclonal antibodies are used to detect the presence of trypanosomes in the suspension. The test is highly specific as monoclonal antibodies are used and it is simple, rapid, and can detect mixed infections via testing of one sample multiple times using different monoclonal antibodies [21, 27, 28]. In the DNA probing technique, a denatured DNA sample (target) fixed on nitrocellulose is exposed to a radioactively labeled DNA-probe, which is a fragment of DNA of variable length. The probe - target complementary base pairing of the sequence in the probe is used to diagnose infection [29–31]. Conventional PCR has also been used [23, 32]. PCR works using either species-specific primers or generalist non-species-specific primers (e.g. ITS1) to differentiate trypanosomes. The advantage of ITS1 PCR is that only one test needs to be done to assess whether trypanosomes occur in the sample –regardless of the species, whereas in the species-specific PCR a sample must be tested repeatedly with each species-specific primer pair [33, 34]. Trypanosome detection by PCR is done using the entire tsetse body or different tsetse organs and recently also even anal and oral droppings are used [35, 36]. Another modern technique is the fluorescent fragment-length barcoding method (FFLB), which is a hybrid of PCR and sequencing. FFLB amplifies fragments with inter-species size variation by PCR using fluorescently tagged primers, then, the sizes of the fragments of the PCR product are determined accurately using an automated DNA sequencer. Therefore, it discriminates trypanosome species by size polymorphisms. However, FFLB is too advanced and expensive for routine use in Africa [14, 15, 33]. Real-time PCR has the inherent ability to detect and quantify the number of trypanosomes in a sample [37]. Finally, loop mediated isothermal amplification (LAMP) is a low-tech trypanosome detection technique. The target sequence is amplified by LAMP at a constant (isothermal) temperature of 60–65 °C using either two or three sets of primers and a polymerase with high strand displacement activity in addition to a replication activity. Typically, 4 different primers are used to identify 6 distinct regions on the target gene, which leads typically to good specificity. The added advantage of the LAMP technique is that it does not require experience nor instruments except a water bath or incubator and results are obtained quickly [38].

## Results

### Inclusion of studies and data extraction

A total of 605 studies were initially screened of which 191 were excluded on the basis of their titles and abstracts. Of the remaining 414 which were fully evaluated, 72 were considered while 342 studies were excluded (Fig. 1). The 72 selected articles involved 23 countries for a total of 236,740 tsetse flies checked for trypanosome infection (Additional file 1). Of those 72 articles, 49 were reporting field studies with 202,182 tsetse flies analysed. The majority of the field studies (80%) used dissection (i.e. on 192,338 tsetse flies in total). The remaining 23 studies were laboratory experiments with 34,558 tsetse flies analysed of which 18 studies used dissection method. The studies included 12 different tsetse species. Samples analysed were saliva spit, anal droppings (diuresis fluid), midgut, proboscis, salivary glands and/or their DNA, DNA and pools of DNA from whole bodies. Methods used for the detection of trypanosomes were: dissection, microscopy of diuresis, probing on mice, warm slide probe, culture using media, isoenzyme analysis, DNA probing, Dot-ELISA, species specific PCR, ITS-1 PCR, real time PCR, species-specific LAMP and FFLB. Details are provided in Tables 1 and 2.

**Table 1:** List of published articles included in the systematic review for field studies with dissection method used for diagnosis of trypanosome infection

Variable	No studies	No species	No flies	References
Overall field studies	49	12^a^	202,182	[8, 9, 14, 15, 27, 29, 30, 33–35, 44–80]
Country				
Angola	1	1	62	[54]
Burkina Faso	1	2	435	[44]
Cameroon	3	5^a^	6104	[12, 77]
Democratic Republic of Congo	1	1	254	[76]
Equatorial Guinea	1	1	62	[35]
Ethiopia	1	4	384	[51]
Gambia	2	4^a^	3055	[30, 61]
Ivory Coast	3	5	3707	[50, 52, 62]
Kenya	7	4^a^	41,959	[13, 27, 29, 45, 71, 78, 79]
Liberia	1	3	2224	[72]
Nigeria	4	3	27,502	[56, 58, 73, 75]
Rwanda	1	3	5496	[65]
South Africa	3	2^a^	1323	[53, 60, 68]
Southern Sudan	1	1	117	[66]
Tanzania	10	4^a^	43,923	[8, 9, 14, 15, 33, 34, 46, 48, 59, 60]
Uganda	3	4	16,350	[55, 74, 80]
Zambia	6	3^a^	49,225	[47, 49, 57, 64, 70, 72]
Detection method				
Culture media	1	3	1112	[62]
Dissection	38	12^a^	192,338	[8, 9, 12, 13, 15, 27, 29, 30, 34, 44–52, 55, 56, 58, 59, 61–63, 65, 67–69, 71–75, 77–80]
Dot-ELISA	1	2	494	[27]
FFLB	1	^a^	91	[14]
ITS-1 PCR	2	1^a^	173	[14, 59]
Sp. specific PCR	11	4^a^	7974	[35, 53, 54, 57, 60, 64, 66, 70, 71, 76, 77]
Glossina sample type				
DNA DO and Pool NDO	1	^a^	3638	[71]
DNA WB	4	3	1221	[54, 57, 64, 66]
DNA WB & DO	1	1	279	[59]
Pool DNA WB	2	1^a^	312	[35, 70]
Mid gut	8	5^a^	20,792	[14, 33, 34, 48, 62, 63, 76, 77]
MP	8	7^a^	46,416	[8, 9, 15, 29, 53, 56, 58, 68]
MS	1	2	1221	[50]
MPS	19	10^a^	73,793	[12, 27, 30, 44, 46, 61, 64, 65, 68, 69, 71–75, 78–80]
Proboscis	5	5^a^	46,991	[13, 47, 49, 60, 67]
SG	1	1	7519	[45]
Glossina species				
*G. austeni*	1	1	40	[68]
*G. brevipalpis*	6	1	5870	[9, 49, 55, 60, 65, 68]
*G. fuscipes*	5	1	7071	[49, 51, 66, 74, 80]
*G. longipennis*	2	1	1305	[27, 71]
*G. medicorum*	1	1	10	[52]
*G. morsitans*	10	1	51,556	[9, 30, 44, 49, 51, 56, 61, 65, 72, 75]
*G. nigrofusca*	3	1	294	[50, 62, 65]
*G. pallicera*	2	1	76	[62, 63]
*G. pallidipes*	20	1	64,395	[8, 9, 13, 15, 27, 29, 45, 47–49, 51, 55, 57, 59, 64, 65, 69, 71, 78, 79]
*G. palpalis*	14	1	14,669	[12, 30, 35, 50, 52, 54, 55, 58, 62, 63, 67, 73, 76, 77]
*G. swynnertoni*	5	1	14,414	[8, 9, 15, 46, 48]
*G. tachinoides*	6	1	6367	[44, 51, 52, 58, 73, 75]
Mixed^a^	9	1	35,865	[12, 14, 15, 30, 33, 34, 49, 53, 71]
Not determined	1	1	250	[70]

^a^= mixed tsetse sp. examined besides the indicated number of tsetse sp., *DNA DO and Pool NDO* DNA of dissected organs and of a pool of negative tsetse organs, *DNA WB* DNA of whole fly body, *DNA WB & DO* DNA of whole fly body and of dissected organs, *Pool DNA WB* pooling DNA of whole fly body, *MP* Mid gut and proboscis, *MS* Mid gut and salivary gland, *MPS* Mid gut, proboscis and salivary gland, *SG* Salivary gland, *SP* Salivary gland and proboscis

**Table 2:** List of published articles included in the systematic review for laboratory experimental studies with dissection method used for diagnosis of trypanosome infection

Variable	No studies	No species	No flies	References
Overall experimental studies	23	7^a^	34,558	[21, 22, 24, 28, 31, 36, 37, 81–97]
Country				
Belgium	3	2	1559	[24, 85, 88]
Burkina Faso	2	1^a^	1443	[36, 87]
BFZ	1	2^a^	1092	[95]
France	1	1	594	[97]
Ghana	2	2	540	[28, 86]
Kenya	5	7	19,592	[21, 31, 81–83]
Tanzania	1	1	3274	[22]
Uganda	2	4	2011	[89, 90]
United Kingdom	4	2	721	[37, 91, 94, 96]
Zambia	1	1	1796	[92]
Zimbabwe	1	1	1936	[93]
Detection method				
Dissection	17	7^a^	22,478	[21, 24, 36, 81–83, 85, 87–95, 97]
DNA probe	1	1	15	[31]
Dot-ELISA	3	2	1240	[21, 28, 86]
Microscopy of diuresis	1	^a^	266	[95]
Sp. Specific PCR	2	3^a^	1246	[21, 95]
probing on mice	1	1	300	[96]
Real Time PCR	1	1	150	[37]
Warm slide probe	5	1^a^	8863	[22, 82, 93–95]
Glossina sample type				
DF	1	^a^	532	[95]
Mid gut	5	2	2205	[28, 31, 37, 92, 94]
MP	3	2	1573	[85, 88, 95]
MS	2	2	1754	[22, 93]
MPS	5	2^a^	4263	[21, 24, 86, 87, 91]
proboscis	3	6	12,315	[81–83]
SS	5	1^a^	9011	[22, 84, 93–95]
SG	2	4	2411	[90, 97]
SP	1	1	194	[89]
N.A	1	1	300	[96]
Glossina species				
*G. austeni*	1	1	1062	[83]
*G. brevipalpis*	2	1	1256	[83, 90]
*G. fuscipes*	2	1	1570	[83, 90]
*G. morsitans*	19	1	22,607	[22, 24, 28, 31, 37, 81–83, 85, 86, 89–97]
*G. pallidipes*	4	1	2619	[21, 86, 90, 94]
*G. palpalis*	4	1	2184	[36, 83, 88, 95]
*G. tachinoides*	1	1	1009	[83]
Mixed^a^			2251	[87, 95]

^a^= mixed tsetse sp. examined besides the indicated number of tsetse sp., *DF* Diuresis fluid, *MP* Mid gut and proboscis, *MS* Mid gut and salivary gland, *MPS* Mid gut, proboscis and salivary gland, *SS* Saliva spit, *SG* Salivary gland, *SP* Salivary gland and proboscis, *N.A.* Not available

One third of the studies compared dissection positive results with at least one alternative serological or molecular technique: species-specific PCR (*n* = 15), DNA probe (*n* = 4), fluorescent fragment length barcoding (*n* = 3), ITS-1 PCR (*n* = 2), and dot-ELISA (*n* = 1).

### Meta-analysis of dissection based field studies

**Fig. 2:**
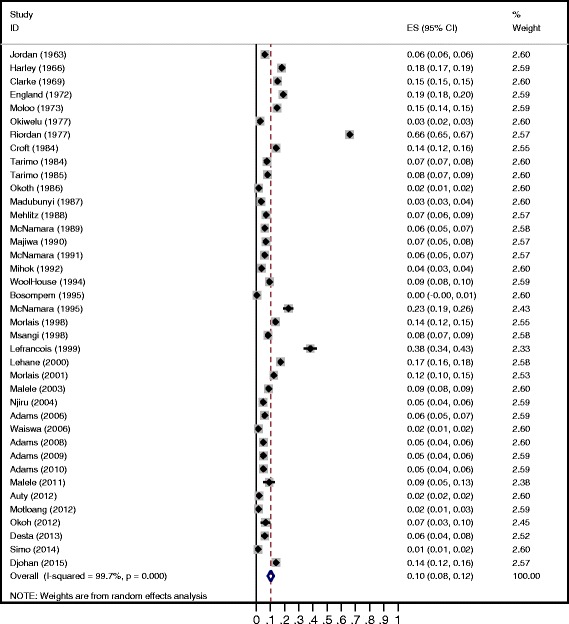
Forest plot of the prevalence of trypanosome infection in tsetse flies by the dissection method for field studies

The overall trypanosome prevalence of flies in the field studies (*n* = 39) was 10.3% (95% CI = 8.1, 12.4) (Fig. 2). Significant between-study heterogeneity was observed (*P* < 0.001). Different factors were thus further analysed. Results are presented in Table 3. The prevalence of trypanosomes decreases with publication year (*P* = 0.035). Trypanosome prevalence differs significantly between countries (*P* = 0.004). The prevalence ranged from 4.1% in Rwanda to 40.5% in Burkina Faso. The type of tsetse fly sample (body part) did not have a significant effect on the prevalence (*P* = 0.2155). The prevalence of trypanosomes ranged from 6.5% in midguts to 30.8% in the pooled midgut/salivary glands samples. Tsetse fly species or group (morsitans, fusca, palpalis) were not significant factors (*P* = 0.1466). The highest trypanosome prevalence was observed in *G. negrofusca* (26%, *n* = 3, fusca group) and the lowest was observed in *G. longipennis* (0.2%, *n* = 2, fusca group). The two variables that were significant in the univariate analysis (Table 3) remained significant with minor changes in the estimates of the odds ratios in the multivariate meta-regression analysis (results not shown).

**Table 3:** Univariate meta-regression analysis of factors for the prevalence of trypanosome infection based on the field studies with dissection method used for diagnosis of trypanosome infection

Variables	Prevalence			Odds ratio			
	Estimate	95% CI		Estimate	95% CI		*p*-value
Year of publication	-		-	0.998	0.996	0.999	0.035
Country							
Burkina Faso	40.5	28.1	53.0	Ref.			
Ivory Coast	18.6	13.7	23.6	0.82	0.69	0.97	0.021
Nigeria	18.0	5.0	30.9	0.80	0.67	0.95	0.010
Cameroon	10.9	2.9	18.8	0.74	0.62	0.89	0.002
Uganda	10.0	3.0	17.0	0.74	0.62	0.88	0.001
Zambia	8.6	3.1	14.1	0.73	0.61	0.86	<0.001
Liberia	8.2	4.4	12.1	0.74	0.61	0.89	0.002
Kenya	7.4	3.5	11.4	0.72	0.61	0.85	<0.001
Tanzania	7.0	5.2	8.9	0.71	0.61	0.84	<0.001
South Africa	6.8	0.2	20.5	0.71	0.58	0.88	0.002
Gambia	6.0	5.2	6.8	0.71	0.59	0.85	<0.001
Ethiopia	4.9	0.6	9.2	0.70	0.58	0.84	<0.001
Rwanda	4.1	3.0	5.1	0.70	0.58	0.84	<0.001
Glossina sample type							
MS	30.8	7.6	53.9	Ref.			
Salivary gland	19.3	18.4	20.2	0.91	0.69	1.19	0.472
MPS	11.2	8.5	14.0	0.84	0.72	0.98	0.023
Proboscis	10.6	4.3	16.9	0.83	0.70	0.98	0.029
Mid gut	6.5	4.6	8.3	0.83	0.70	0.98	0.027
Mid gut and proboscis	6.5	5.0	8.0	0.80	0.68	0.94	0.007
Glossina species							
*G. nigrofusca*	25.9	1.9	49.9	Ref.			
*G. pallicera*	23.5	12.4	34.7	1.04	0.81	1.34	0.751
*G. medicorum*	20.0	4.8	44.8	0.96	0.66	1.39	0.811
*G. tachinoides*	17.7	8.5	26.9	0.93	0.78	1.12	0.454
*G. morsitans*	15.8	9.0	22.6	0.92	0.77	1.08	0.304
*G. austeni*	15.0	3.9	2.16	0.91	0.68	1.22	0.520
*G. palpalis*	10.5	6.4	14.6	0.87	0.74	1.03	0.107
*G.* mixed species	9.6	4.3	14.8	0.86	0.72	1.03	0.102
*G. swynnertoni*	9.2	3.0	15.4	0.86	0.71	1.03	0.101
*G. pallidipes*	8.3	5.8	10.8	0.85	0.72	1.00	0.052
*G. brevipalpis*	5.8	2.3	9.3	0.83	0.69	1.00	0.046
*G. fuscipes*	1.1	0.1	2.1	0.79	0.65	0.96	0.017
*G. longipennis*	0.2	0.1	0.5	0.78	0.63	0.98	0.031

*MS* Mid gut and salivary gland, *MPS* Mid gut, proboscis and salivary gland

### Meta-analysis of dissection based laboratory experiments

**Fig. 3:**
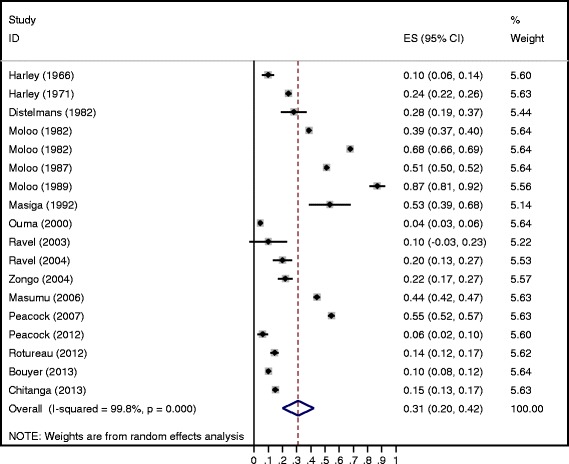
Forest plot of the prevalence of trypanosome infection in tsetse flies by the dissection method for laboratory experiments

The overall trypanosome prevalence of flies in the laboratory experiments (*n* = 18) was 31.0% (95% CI = 20.0, 42.0) (Fig. 3). Significant between-study heterogeneity was also observed in these studies (*P* < 0.001). However, the trypanosome prevalence did not differ significantly between countries (*P* = 0.0916) nor as a function of the publication year (*P* = 0.184) (Table 4). No significant (*P* = 0.9545) differences in trypanosome prevalence among the seven tsetse species were observed. The sample type (body part) was significantly (*P* = 0.0122) associated with trypanosome prevalence. The highest trypanosome prevalence was observed in the proboscis.

**Table 4:** Univariate meta-regression analysis of factors for the prevalence of trypanosome infection based on the laboratory experimental studies with dissection method used for diagnosis of trypanosome infection

Variables	Prevalence (%)			Odds ratio			
	Estimate	95% CI		Estimate	95% CI		*p*-value
Year of publication	-		-	0.996	0.99	1.00	0.184
Country							
Zimbabwe	54.5	52.2	56.8	Ref			
Kenya	50.9	31.7	70.0	0.96	0.62	1.51	0.866
Zambia	44.3	42.0	46.6	0.90	0.49	1.65	0.726
Uganda	21.4	14.9	27.9	0.71	0.45	1.15	0.155
Belgium	20.8	13.9	27.8	0.72	0.44	1.18	0.181
United Kingdom	20.5	0.0	41.3	0.71	0.43	1.17	0.171
Burkina Faso and Zimbabwe	20.1	9.1	31.0	0.71	0.42	1.22	0.192
France	14.5	11.6	17.3	0.67	0.37	1.22	0.181
Burkina Faso	10.0	8.4	11.5	0.64	0.38	1.08	0.093
Glossina sample type							
Proboscis	56.1	43.3	68.8	Ref			
Mid gut and salivary gland	32.7	0.0	76.3	0.80	0.59	1.09	0.145
Salivary gland	22.3	16.8	27.8	0.71	0.58	0.89	0.004
Mid gut and proboscis	20.2	13.5	26.9	0.70	0.56	0.89	0.005
Mid gut	19.0	0.0	51.4	0.69	0.53	0.89	0.007
MPS	17.6	10.1	25.1	0.71	0.56	0.89	0.006
Salivary gland and proboscis	9.8	5.6	14.0	0.63	0.42	0.95	0.028
Glossina species							
*G. brevipalpis*	51.9	0.0	100.0	Ref			
*G. tachinoides*	40.2	37.2	43.3	0.89	0.48	1.66	0.698
*G. morsitans*	37.9	23.6	52.2	0.87	0.59	1.28	0.455
*G. fuscipes*	31.7	11.2	52.2	0.82	0.49	1.36	0.415
*G. austeni*	31.5	28.7	34.2	0.81	0.44	1.52	0.499
*G. palpalis*	31.0	23.4	38.7	0.796	0.52	1.22	0.278
*G. pallidipes*	11.9	0.0	24.0	0.67	0.42	1.07	0.088
*Mixed*	10.0	8.4	11.5	0.66	0.35	1.22	0.174

*MPS* Mid gut, proboscis and salivary gland

### Sensitivity of advanced detection methods

**Fig. 4:**
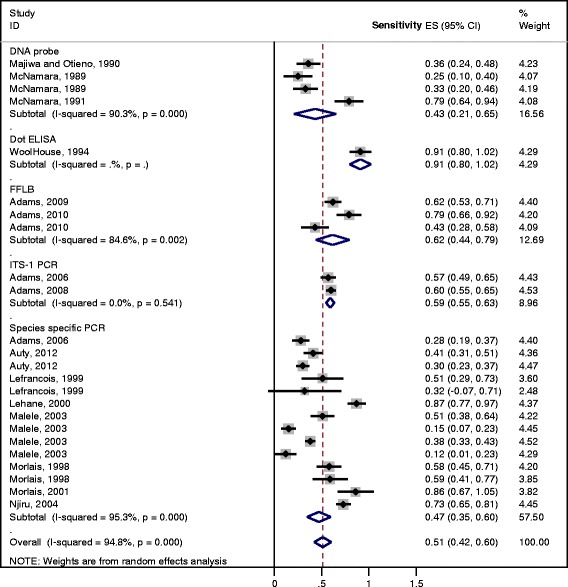
Forest plot for the sensitivity of molecular and serological detection methods of trypanosome infection in the tsetse flies using the dissection method as gold standard

The results of the meta-analysis of the 25 studies using alternative diagnostic tests on dissection positive samples are shown in Fig. 4. With the exception of the dot-ELISA (sensitivity of 91%, which was represented only by one study), the remaining methods had similar levels of sensitivity ranging from 43% for DNA probe to 62% for fluorescent fragment length barcoding.

## Discussion

The scientific literature on trypanosome detection methods and prevalence in tsetse flies published in English since more than a half century covering natural and experimental infections of tsetse flies was reviewed in this paper.

As expected, the prevalence of trypanosome infection in tsetse flies is higher in laboratory experiments than in field collections of tsetse flies with an overall prevalence of 31% and 10% respectively. In laboratory experiments, blood meals and external conditions are standardized and all feeding flies ingest parasites. In field collected tsetse flies, the prevalence of trypanosome infected hosts and their parasitaemia will be the determining factors explaining the prevalence in flies.

From our meta-analysis, it appears that differences in prevalence exist in field collected tsetse flies according to year and country. The factor “country” should be interpreted with care as many factors can explain the differences between countries, e.g., the ecological context and national vector control measures. However, original studies didn’t investigate the real ecological, entomological, parasitological, tsetse-host contact and intervention factors to identify the actual risk factors responsible for the infection of tsetse flies. If our review allowed pinpointing spatial differences, it could not clearly bring a precise explanation of the variations in infection prevalence.

The negative relationship between the year of publication and the prevalence of infection is a paradox. Encroachment, i.e., the degrading effect of human activities on the environment, has taken place in most regions of sub-Saharan Africa. Encroachment causes a decrease of the tsetse fly population size. However, as counterbalancing effect, the prevalence of the trypanosome infection of the flies has been observed to increase allowing for persistent transmission even when the tsetse fly vectors are scarce [39, 40]. However, the opposite effect is observed. This might be due to the higher intensity of drug treatment of the livestock by the farmers. Indeed, most farmers in endemic areas treat their herds regularly using trypanocidal drugs [41]. It is known that prolonged and persistent use of trypanocidal drugs in the field decreases/disrupts the transmission of trypanosomes by the tsetse flies [42, 43] thus reducing the risk of tsetse infection.

Our last objective was to assess the sensitivity of various diagnostic methods for the detection of trypanosomes in the tsetse flies using the dissection method as gold standard. The sensitivity of molecular/serological tests that were performed on positive samples (i.e. by dissection) was only around 50%. The alternative diagnostic tools applied to the dissection positive samples were thus characterised by low sensitivity, and no information on specificity is available at all. The currently available molecular and serological techniques are developed and optimized for trypanosome detection in the host; their detection performance in the insect (tsetse fly) is a different story. This study revealed that the tests apparently work suboptimal for tsetse fly samples. Sample processing conditions and specimens used are not standardized or externally controlled for detection of trypanosomes in tsetse flies. Comparing several tests on the same specimen panel would allow more accurate comparisons of the sensitivity and specificity.

## Conclusions

Dissection remains the gold standard for the determination of the infection status of tsetse flies. Alternative molecular and serological techniques have currently too low sensitivity and their specificity is unknown, which warrants further investigation before they can be employed on a routine basis. Both temporal and spatial variation in trypanosome infection prevalence of field collected tsetse flies exists, but it needs to be investigated further how this variation can be explained by thorough real risk factor investigation for tsetse fly infection.

## Additional file

Additional file 1: Raw data for meta-analysis. (XLSX 14 kb)

## Abbreviations

FFLB: Fluorescent fragment-length barcoding
LAMP: Loop mediated isothermal amplification
PRISMA: Preferred Reporting Items for Systematic Reviews and Meta-Analyses
